# Assessment of the key regulatory genes and their Interologs for Turner Syndrome employing network approach

**DOI:** 10.1038/s41598-018-28375-0

**Published:** 2018-07-04

**Authors:** Anam Farooqui, Safia Tazyeen, Mohd. Murshad Ahmed, Aftab Alam, Shahnawaz Ali, Md. Zubbair Malik, Sher Ali, Romana Ishrat

**Affiliations:** Centre for Interdisciplinary Research in Basic Sciences, Jamia Millia Islamia, New Delhi, 110025 India

## Abstract

Turner Syndrome (TS) is a condition where several genes are affected but the molecular mechanism remains unknown. Identifying the genes that regulate the TS network is one of the main challenges in understanding its aetiology. Here, we studied the regulatory network from manually curated genes reported in the literature and identified essential proteins involved in TS. The power-law distribution analysis showed that TS network carries scale-free hierarchical fractal attributes. This organization of the network maintained the self-ruled constitution of nodes at various levels without having centrality–lethality control systems. Out of twenty-seven genes culminating into leading hubs in the network, we identified two key regulators (KRs) i.e. KDM6A and BDNF. These KRs serve as the backbone for all the network activities. Removal of KRs does not cause its breakdown, rather a change in the topological properties was observed. Since essential proteins are evolutionarily conserved, the orthologs of selected interacting proteins in C. *elegans*, cat and macaque monkey (lower to higher level organisms) were identified. We deciphered three important interologs i.e. KDM6A-WDR5, KDM6A-ASH2L and WDR5-ASH2L that form a triangular motif. In conclusion, these KRs and identified interologs are expected to regulate the TS network signifying their biological importance.

## Introduction

Search of disease related genes has gained momentum during the past one decade. Perhaps this is due to ever growing newer diseases on the horizon or perhaps a drastic change in the lifestyle of the people or both. Finding the cure of the disease requires its identification and diagnosis well on time. Though an increase in discovering disease-associated genes have been observed with time, there is still a large fraction of diseases without a known molecular basis. Network-based analysis of proteins has attained ample attention in recent years. Network-based approaches serve as potent prognostic tools that have been successfully applied in the characterization of genes in complex diseases such as cancer, ataxia, multiple sclerosis etc.^1–4^. Understanding gene regulatory network advances our knowledge regarding initiation and progression of disease. Such studies will augment system biology research enhancing the efficacy of different therapeutic approaches.

Turner Syndrome (TS) is one such condition where a partial knowledge of its molecular basis is known but a large proportion of their associated candidate genes are still unknown. It is a rare chromosomal disorder affecting females where an X chromosome of a female is partly or completely missing due to sporadic chromosomal non-disjunction^5^. This results in XO condition. Besides the cases of monosomy X (45, X) being the most common in TS, several cases have also been reported with mosaicism, where 45, X cell line is accompanied by one or more other cell lines having a complete or structurally abnormal sex chromosomes (X or Y)^6^. Signs and symptoms of TS are highly variable differing dramatically from one person to another. Thus, the association between genotype and phenotype also remains a challenge. It is known that mosaic cases show up milder phenotypic anomalies compared to those with 45, X karyotype. Of all the observed symptoms, short stature and gonadal dysgenesis remains the most consistent one^6^. The other phenotypes of TS can be associated to the features that are less frequent such as cardiovascular congenital defects, aorta anomalies, renal alterations, cognitive inability that includes selective non-verbal deficiencies etc. Mental deficiency is not a characteristic of TS. It is believed that additional, as-yet-unidentified genes on the X chromosome and some autosomal genes may play a role in the development of these co morbidities of TS. Identification of these factors is still lacking and needs to be evaluated.

The current approach for the prioritization of disease genes is mainly centred on the ‘guilt-by-association’ assumption, which means that the physically and functionally related genes are likely to be involved in the same biological pathways having comparable effects on the phenotypes^7^. Network theory is an important approach to understand topological properties and the dynamics of complex systems to co-relate with their functional modules. Most of the existing networks may be categorised into one of them, namely, scale-free, small world, random and hierarchical network. Amongst them, hierarchical type of network is of special interest to biologists as it includes the appearance of modules and sparsely distributed hubs regulate the network.

Based on this understanding, the key regulators of the TS were identified by integrating protein-protein interaction (PPI) network in the present study. Our current paradigm for studying TS revolves around the identification of the key regulators of TS among manually curated genes by combining protein interactions, functions, disease networks and orthologs. We also aim to understand its topological properties to predict important key regulators among which some are of fundamental importance for their activities and regulating mechanism.

## Results

### Data mining and curation of genes related to Turner Syndrome

Through literature search, a list of thirty-one genes reportedly involved in TS and its related comorbidities was obtained (Table 1). These genes will be used for network construction and to study their biological significance.

**Table 1:** List of manually curated genes involved in TS.

SN	Gene Name	Description	Location	References
1)	**SHOX**	Short Stature Homeobox	Xp22.33 and Yp11.2	^5, 35^
2)	SRY	Sex-determining Region Y	Yp11.2	^36, 37^
3)	**KDM6A**	Lysine Demethylase 6 A	Xp11.3	^12, 38^
4)	TSPY1	Testis specific protein, Y-linked 1	Yp11.2	^39^
5)	**RPS4X**	Ribosomal protein S4, X-linked	Xq13.1	^40, 41^
6)	RPS4Y1	Ribosomal protein S4, Y-linked	Yp11.2	^42^
7)	**CSF2RA**	Colony Stimulating Factor 2 Receptor Alpha	Xp22.33 and Yp11.2	^43, 44^
8)	**PRKX**	Protein Kinase, X linked	Xp22.33	^12, 45^
9)	ZFYVE9	Zinc finger FYVE domain-containing protein 9	1p32.3	^12^
10)	**TIMP1**	TIMP metallopeptidase inhibitor 1	Xp11.3	^12, 46^
11)	IGF1	Insulin-like growth factor 1	12q23.2	^47^
12)	**STS**	Steroid Sulphate	Xp22.31	^43, 48^
13)	**NLGN4X**	Neuroligin 4, X-Linked	Xp22.32-p22.31	^48, 49^
14)	MTHFR	Methylenetetrahydrofolate reductase	1p36.22	^50^
15)	GHR	Growth Hormone Receptor	5p13.1-p12	^51^
16)	BDNF	Brain derived Neurotrophic Factor	11p14.1	^13^
17)	VDR	Vitamin D (1,25- dihydroxyvitamin D3) receptor	12q13.11	^52^
18)	AR	Androgen Receptor	Xq12	^53^
19)	FOXP3	Forkhead box P3	Xp11.23	^54^
20)	KCNH2	Potassium voltage-gated channel subfamily H member 2	7q36.1	^55^
21)	SCN5A	Sodium voltage-gated channel alpha subunit 5	3p22.2	^55^
22)	IGFBP3	Insulin like growth factor binding protein 3	7p12.3	^56^
23)	PTPN22	Protein Tyrosine Phosphatase, non-receptor type 22	1p13.2	^57^
24)	XIAP	X-Linked Inhibitor of Apoptosis	Xq25	^58^
25)	AMH	Anti-Müllerian Hormone	19p13.3	^59^
26)	PTPN1	Protein Tyrosine Phosphatase, Non-Receptor Type 1	20q13.13	^60^
27)	DAZ1	Deleted in azoospermia 1	Yq11.223	^36^
28)	**USP9X**	Ubiquitin Specific Peptidase 9, X-Linked	Xp11.4	^48, 61^
29)	**TMEM27**	Transmembrane protein 27	Xp22.2	^48, 62^
30)	**EFHC2**	EF-Hand Domain Containing 2	Xp11.3	^63^
31)	SOCS2	Suppressor Of Cytokine Signalling 2	12q22	^56^

^*^The dosage sensitive X linked genes are highlighted in bold.

### Turner Syndrome network follows hierarchical scale free features

**Figure 1:**
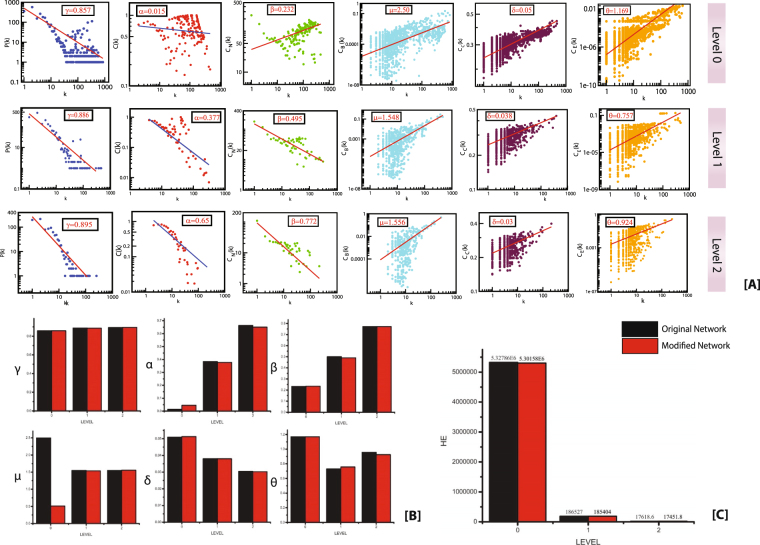
(**A**) The behaviours of degree distributions (P(k)), clustering co-efficient (C(k)), neighborhood connectivity (C_N_(k)), betweenness (C_B_(k)), closeness (C_C_(k)) and eigen-vector (C_E_(k)) measurements as a function of degree k for original and BDNF knockout network at different levels of organization. (**B**) The changes in the exponents of the six topological parameters due to BDNF knock-out experiment. (**C**) Changes in the Energy distribution in the network quantified by Hamiltonian calculation as a function of network levels in original and BDNF knockout network.

**Figure 2:**
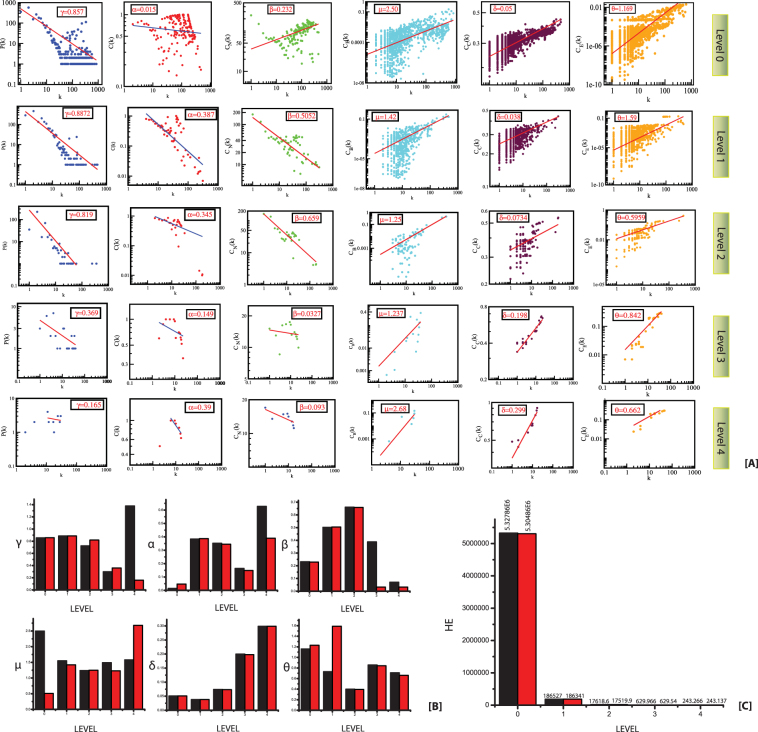
(**A**) The behaviours of degree distributions (P(k)), clustering co-efficient (C(k)), neighborhood connectivity(C_N_(k)), betweenness (C_B_(k)), closeness (C_C_(k)) and eigen-vector(C_E_(k)) measurements as a function of degree k for original and KDM6A knockout network at different levels of organization. (**B**) The changes in the exponents of the six topological parameters due to KDM6A knock-out experiment. (**C**) Changes in the Energy distribution in the network quantified by Hamiltonian calculation as a function of network levels in original and KDM6A knockout network.

The candidate genes listed in Table 1 were used to construct their regulatory network of which only twenty-seven genes participated as leading hubs. The main constructed network consisted of 3294 nodes and 97361 edges. The topological properties used to characterise the structural and organizational topographies of the TS network are probability of degree distribution P(k), clustering co-efficient C(k) and neighborhood connectivity C_N_(k). These properties could perhaps relate to the functional and self-similar (fractal) constituents of the network. It was observed that these topological properties obey power law behaviour as a function of degree k (Figs 1(A) and 2(A) first row against Level 0). The power law fits on the data sets of the topological variables of the network are done and verified following a standard statistical fitting procedure proposed by Clauset *et al*.^8^. Here, all statistical p-values for all data sets, calculated against 2500 random samplings, are found to be larger than a critical value 0.1, and goodness of fits is found to be less than and equal to 0.33. The values of the exponents are obtained from the power law fittings. The results for the complete network are summarized as follows,

1 $$[\begin{array}{c}P\\ C\\ {C}_{N}\end{array}]\sim [\begin{array}{c}{k}^{-\gamma }\\ {k}^{-\alpha }\\ {k}^{+\beta }\end{array}]\to [\begin{array}{c}0.857\\ 0.015\\ 0.232\end{array}]$$

The negative values of α and γ suggest that the TS network follows hierarchical nature. The value of $\gamma =\,\mathrm{ln}(2.6)/\mathrm{ln}(3)$ which is 0.857, means that number of nodes increase with the advancement of disease as a power of 2.6 while links as a power of 3 thus, giving us the idea of being hierarchical as it shows presence of modules in our clustering experiment. The positive value of β indicates that the network carries the assortive mixing specifying that a large cluster of degree nodes (formation of rich club) regulates the TS network.

The centrality measurements, namely betweenness centrality C_B_(k) and closeness centrality C_C_(k) represent the flow of information in the network and predict the influential candidates in the network. The well connectedness of nodes in a network is characterised by eigenvector centrality C_E_(k). It measures the efficacy of the spreading (receiving) power of information of nodes from the network. These properties obey power law behaviours as follows,

2 $$[\begin{array}{c}{C}_{B}\\ {C}_{C}\\ {C}_{E}\end{array}]\sim [\begin{array}{c}{k}^{\mu }\\ {k}^{\delta }\\ {k}^{\theta }\end{array}]\to [\begin{array}{c}2.50\\ 0.051\\ 1.16\end{array}]$$

The power law natures of the three centrality measurements are again verified and confirmed using the procedure of Clauset *et al*.^8^ of statistical power law fitting. Here p values are found to be more than 0.1 and goodness of fit larger than 3.5. It was observed that only few numbers of higher degree nodes have large centrality values (for all three centrality measurements). The number of most influencing hubs, which can control the network, is few. Therefore, the TS network is dominated by the low degree nodes (genes/proteins) and these low degree nodes regulate the functioning and organization of the network. Few of the sparsely distributed leading hubs might, however, take important participation in regulating as well as maintaining the network stability. The positive values of these centrality measurements show that the network exhibits hierarchical scale free or fractal features.

Thus, the overall topological properties of the TS network indicate that the same self-organise into a scale free fractal state and have hierarchical organization, i.e. they are composed of successive interconnected or inter-nested communities.

### Identification of key regulators and properties

**Figure 3:**
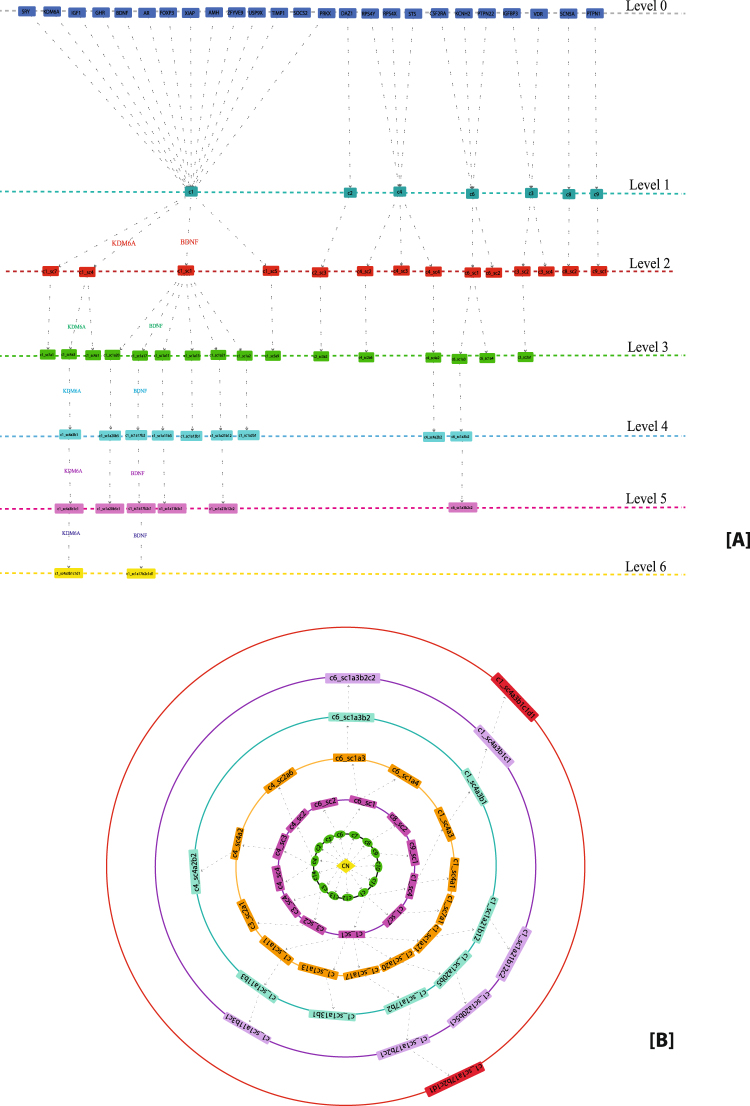
(**A**) Tracing of KRs through different levels in the network. (**B**) Organization of the modules/sub-modules of the network.

**Figure 4:**
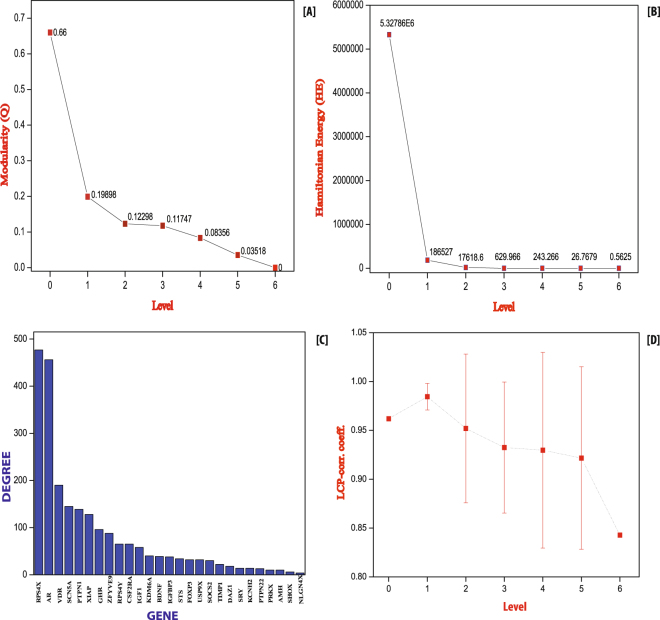
(**A**) Corresponding modularity Q_N_ as a function of levels of organization. (**B**) Corresponding Hamiltonian Energy (HE) as a function of levels of organization. (**C**) Characterization of twenty-seven leading hubs of the network by degree (**D**). Variation in the calculated average LCP-corr for TS network as a function of network level.

The modular structure and their arrangement at various levels of organization are done following Newman and Girvan’s standard community finding algorithm^9^. Using this algorithm, the TS network is found to be hierarchically organised through six different levels (Fig. 3(B)). The corresponding modularity Q_N_ and Hamiltonian Energy (HE) as a function of levels of organization are found to be decreased as one goes from top to down organisation (Fig. 4(A,B), respectively).

**Figure 5:**
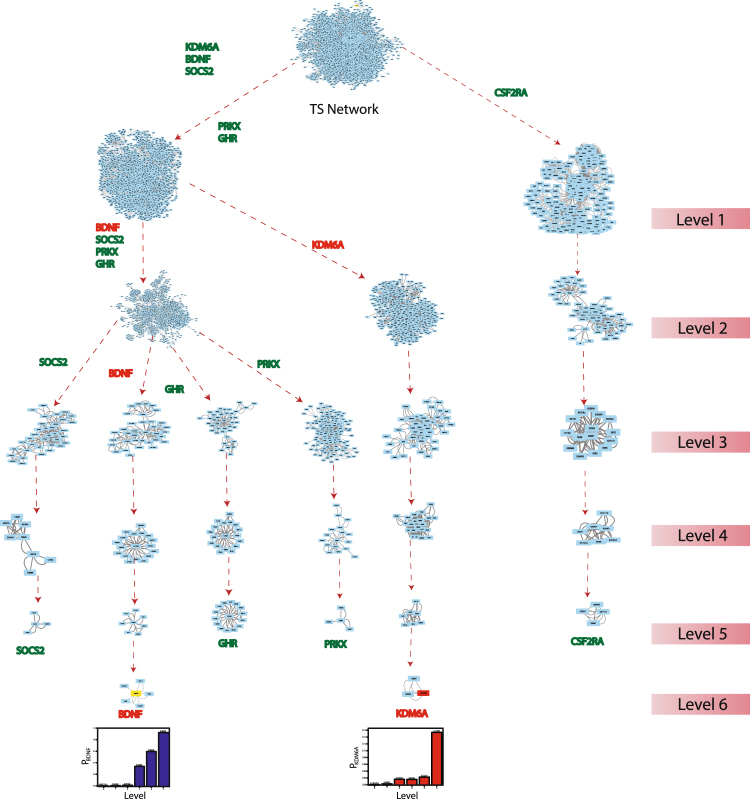
Network/modules/sub-modules at different network levels which accommodate leading hubs and key regulators. The probability distribution of the KRs as a function of level.

Here we put forward the idea of *key regulators* (KRs) as the genes/proteins which are deeply rooted from top to bottom of the network and vice versa, which serve as the backbone of the network organization. It is not essential for these KRs to be the large leading hubs in the network, but they rather change their popularities randomly at various levels of organization (Fig. 5). Since the network qualifies hierarchical characteristics, the removal of the leading hubs will not cause its breakdown. However, the removal of KRs from the network may cause maximum local and global perturbations, especially at a deeper level of organization. These perturbations will propagate through various levels of organization’s bottom to top or top to bottom causing topological change in the network. Thus, these KRs could be the possible key target genes of the TS network.

Following the definition of KR, we identified two KRs, namely, KDM6A and BDNF (Fig. 5), which are key regulators/organizers of the TS network. Unexpectedly, the first eleven leading hubs are not found to be KRs since they fail to reach till the deepest level of organization (Figs 4(C) and 5). These two KRs maintain a low profile/popularity thereby regulating the network till the bottom level of organization. These KRs separate from each other after level one and then move separately till the motif level (Fig. 3(A)). These KRs may act as signal propagators from top to bottom and vice versa to maintain network stability and inherent properties. The proteins SOCS2, GHR, CSF2RA and PRKX move till fifth level but fail to reach the motif level (Fig. 5).

To understand the regulating ability of each of the 2 KRs, we calculated the Probability *P*_*x*_(y^l^) of KR (Fig. 5).

3 $${{\boldsymbol{P}}}_{{\boldsymbol{x}}}({{\boldsymbol{y}}}^{[{\boldsymbol{l}}]})=\,\frac{{{\boldsymbol{y}}}^{[{\boldsymbol{l}}]}}{{{\boldsymbol{E}}}^{[{\boldsymbol{l}}]}}$$

Where x is number of edges *y*^*[l]*^ at level l and *E*^*[l]*^ is total number of edges of the network or modules and sub-modules. The calculated Probability ${P}_{x}({y}^{l})$ of all the key genes show an increase in P_x_ as one goes top to bottom direction as level l increases. This means the regulating ability of each key gene becomes more important at deeper level of organization.

### Evidence of self-organization: local-community-paradigm (LCP) approach

The LCP architecture assists not only the rapid delivery of information across the various network modules, but also through the local processing. We analysed the TS network to check the maintenance of its self-organization at various levels of network organization using LCP technique. The LCP-corr of all the modules/sub-modules at various levels was calculated. The average values of LCP-corr at each level (modules having zero LCP-corr are not taken in average) are greater than 0.85 and the values do not change with error bar (Fig. 4(D)). This indicates that the network maintains self-organisation and is compact and has efficient information processing. It represents strong LCP networks that are dynamic and heterogeneous, which facilitate network evolution and reorganization.

### Local perturbations driven by key regulators

The knock-out experiment of the KRs from the TS network highlights the local perturbations driven and their consequence on global network properties. The knockout experiment for both the KRs was performed separately. In both the cases a significant change in the topological properties of the network is observed (Figs 1(A) and 2(A)). It was seen that α change significantly at level 0, whereas β and γ change only slightly (Figs 1(B) and 2(B)). Similarly, the changes in the exponents of centrality measurements (δ, µ and θ) at level 0 also show significant change (Figs 1(B) and 2(B)). The values of δ and θ changes slightly whereas a significant change is observed in the value of µ. It is evident from the changes in the exponents of topological parameters that as one goes to deeper level i.e. top to down direction the network perturbation increases (Figs 1(B) and 2(B)). In case of KDM6A, after the fourth level, its removal almost breaks down the sub-modules present in the remaining deeper levels. Whereas in case of BDNF, after the second level, its removal almost breaks down the sub-modules present in the remaining deeper levels. This indicates that local perturbation is maximum at deeper levels starting from bottom to top.

We then calculate Hamiltonian energy of the respective complete network and modules/sub-modules in the KR knockout experiment to understand change in energy distributions in the respective network. A slight decrease in the Hamiltonian energy is observed at each level due to knockout of the KRs (Figs 1(C) and 2(C)). This indicates that the removal of KRs cause enormous loss of wiring/rewiring energy which is propagated throughout the levels of network organization.

### Centrality-Lethality in Turner Syndrome network

The TS network is close to hierarchical network and hence the modules/sub-modules emerged are compact at upper levels of organization. The knockout of KRs (KDM6A and BDNF) from TS network does not cause the network breakdown. As observed in case of KDM6A and BDNF, their knockout almost breaks down the sub-modules from fourth and second level respectively, whereas other modules/sub-modules remain stable to preserve the network properties. Hence, TS network rules out centrality-lethality rule^10^. However, the identified KRs have important regulating activities in the network which is reflected in the changes in the topological properties and other parameters of the network and its associated communities at various levels of organization.

### Predicting essential protein interactions: Interologs

Following the centrality measurements-based methodology (see in Methodology), we examined the top thirty genes each one identified by its centrality and degree measurements. We got 120 genes from all measurements (Supplementary Table S1). Among these 120 central genes, 2 genes (HDAC3 and RPS3) were found to be the interacting partners of the hub genes, SRY and RPS4Y respectively in TS network.

Analysing the interacting partners of the disease associated genes play an important role in the prediction of genotype-phenotype associations and helps in identifying new disease genes candidates (i.e. the genes coding for the interacting proteins are putative disease-causing genes). It is thus expected that the interacting partners of the key regulators may also be associated with TS. Built on this assumption 8 PPI were selected for further analysis listed in Table 2.

**Table 2:** Essential PPI interactions in TS network.

Hub Gene	Interacting Partner	Location of Interacting partner
SRY	HDAC3 (Histone Deacetylase 3)	5q31.3
RPS4Y1	RPS3 (40S ribosomal protein S3)	11q13.4
**KDM6A**	**WDR5 (WD repeat-containing protein 5)**	**9q34.2**
	**ASH2L (Ash2 histone methyltransferase complex subunit ASH2)**	**8p11.23**
BDNF	MBTP1 (Membrane-bound transcription factor site-1 protease)	16q23.3-q24.1
	CAPS2 (Calcium-dependent secretion activator 2)	12q21.1-q21.2
	CPE (Carboxypeptidase E)	4q32.3
	NOS3 (Nitric oxide synthase 3, endothelial)	7q36.1

One of the most important features of essential proteins is their conservative property. Many studies have shown that essential proteins evolve much slower than the other proteins. They are more evolutionarily conserved than non-essential proteins^11^. By considering the facts that essential proteins depend not only on the interactions between proteins but also their orthologous properties, we find orthologs of the proteins listed in Table 2 in different species namely, C. *elegans*, cat and macaque monkey (Supplementary Table S2). If two proteins physically interact in one species and they have orthologous counterparts in another species, it is likely that their orthologs interact in that species too. If such conserved interactions exist, they are called interologs.

**Figure 6:**
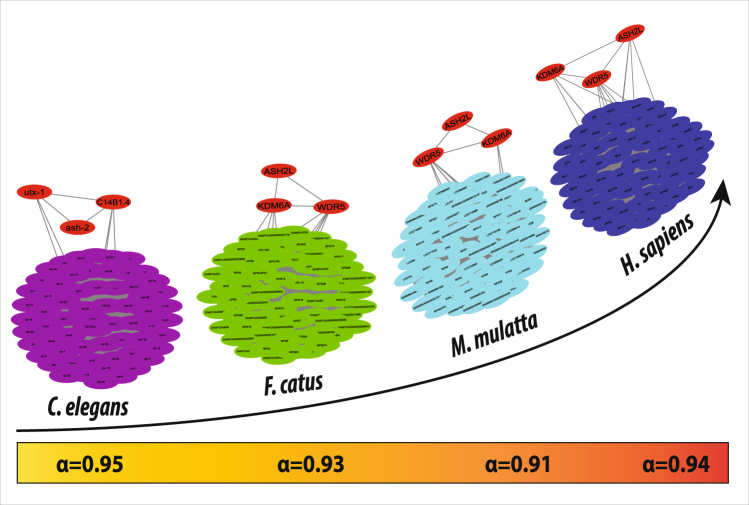
Interologs in the network from lower to higher organism. α is the clustering coefficient of the network.

The network of C. *elegans*, cat, macaque monkey, and human were constructed from these 8 essential interacting proteins as hub genes for the analysis of the interologs (Fig. 6). Though it is expected that all these interactions play important role in TS network regulation, it was observed that only two protein-protein interactions (highlighted in bold in Table 2) remained conserved at each level of organism. Also, it is noteworthy that both these interactions involve the key regulator KDM6A and these proteins form a triangular motif (Fig. 6). These predicted interologs might play major role in the pathophysiology of TS.

## Discussion

Understanding the regulation of a disease network is of great relevance towards the development of treatments for various diseases in the field of pharmacogenomics. One of the ways is to identify the key drivers that regulate the complete network. We have attempted to construct TS network focusing on genes that are regulated through this network.

The TS network constructed from manually curated set of genes show hierarchical features, which means that the network has system level organization involving modules/communities which are interrelated. Since the network is hierarchical, individual gene activities are not of much significance, rather their synchronisation exhibits various important functional regulation of the network. Significant genes (leading hubs) were recognized as key regulators of the network by influencing motifs and module regulation, indicating their biological significance. The leading hubs have significantly important functions. They integrate the lower degree nodes for organizing and regulating activities like inter and intra cross-talk among various other essential genes, maintaining network properties and stability, and optimizing the network signal processing. However, out of these leading hubs, two are key regulators, playing important roles in keeping network in order. In TS network, out of twenty-seven leading hubs, we identified two such KRs which are KDM6A and BDNF. KDM6A is an X-linked gene that plays a central role in coding the histones. It escapes X-chromosome inactivation suggesting that it could be a potential candidate gene of TS^12^. Also, it has been reported that this gene may be involved in premature ovarian failure. BDNF gene is localised on chromosome 11 and is a member of the neurotrophin family of growth factors. It has been reported that TS patients have higher BDNF levels than healthy ones^13^. These KRs serve as the backbone for any network activities and their regulations and could be a possible target gene for disease control. Surprisingly, these KRs do not fall in first few largest hubs (eleven hubs) and thus keep a low profile in the network. Since the network possesses hierarchical properties, removal of KDM6A and BDNF does not cause network breakdown, instead the network adapt itself functionally.

The topological properties of the network exhibit power law behaviour indicating that the network obeys fractal nature. This could be a signature of self-organization in the network. It was also studied that TS represents strong LCP networks that are dynamic and heterogeneous, which facilitate its evolution and reorganization. This indicates that the network maintains self-organisation and is compact and has efficient information processing.

The knock out experiment of the KR indicates that the change in the network properties do not cause significant change in its topology. This indicates that the system does not prefer a change due to perturbation imparted by KR knock out. The network reorganizes itself and adapt according to the changed topological properties. The ability to adapt for a better network organization without breakdown of the system is another signature of self-organization^14^. By considering the facts that essential proteins are more evolutionarily conserved than nonessential ones and essential proteins frequently bind each other, we predicted the essential proteins by integrating the orthology with PPI. We deciphered three important interologs (evolutionarily conserved protein-protein interactions) i.e. KDM6A-WDR5, KDM6A-ASH2L and WDR5-ASH2L, thus forming a triangular motif. KDM6A already has been found to be the key regulator of TS network, therefore these interologs are expected to play major role in TS.

It is likely that the above mentioned genotypic constitution is operative in normal female. However, the Table 1 shows presence of several Y-linked genes including SRY. This raises a question if this scenario is applicable to males also. Alternatively, this may be operative in both the sexes for normal function of both the genome. While this seems to be simple and more convincing proposition, it raises yet another question as we know no two turner patients are alike. Thus, genotypically every Turner is unique with respect to its genotype. If we superimpose on different phenotype of Turner patient, will that eventually generate a consensus on regulatory protein and their interacting partner. While this may be a logical expectation, it still poses yet another challenge both logistical and experimental ones. It would be extraordinarily informative if every single gene listed in Table 1 is analysed with respect to its normal vs mutational status, copy number variation, expressional dynamics and interactomes involved therein. Such analysis would surely augment the understanding on the mechanism of formation of TS and perhaps identification of key regulatory genes. Information on these lines is envisaged to be useful for a better diagnosis and prognosis of TS.

## Methodology

### Data mining and curation of genes related to Turner Syndrome

The information of TS related genes and proteins were collected from the literatures. The genes expected to be involved in TS and related co-morbidities were manually curated from various sources like repositories (PIR)^15^, reviewed literatures (i.e. PubMed), OMIM^16^ etc.

### Construction of Gene Regulatory Network

For the construction of the primary network of the expressed proteins, the curated genes were mapped to their respective UniProt IDs and their associated functional information were retrieved. The simple concept of one gene, one protein was used to build the gene regulatory network of TS.

The network was constructed with available large-scale protein interaction networks using Pathway Commons^17,18^ (a built-in cytoscape feature) and then visualised in cytoscape 3.4 version^19^. Pathway Commons is a metasearch platform, which, in addition to PPI data retrieval, also collects pathway data from multiple publicly available databases, including REACTOME, Systems Biology Centre New York (http://sbcny.org/data.htm), HumanCyc (http://humancyc.org/), The Cancer Cell Map (cancer.cellmap.org) and PID. Pathway Commons includes biochemical reactions, complex assembly, transport and catalytic events and physical interactions. It can easily be accessed either directly online, or through Cytoscape’s built-in import “Network from Web Services” function.

### Characterization of Topological Properties of Networks

The structural properties of complex networks are characterized through the behaviours of the topological parameters. The following topological properties of the networks (graph) were studied to learn the important behaviours of the network: Degree distribution, Neighborhood connectivity, clustering co-efficient, Betweenness centrality, Closeness centrality and Eigenvector centrality.

#### Degree distribution

In a network, the degree k is a centrality measure that represents the number of links the node connects with other nodes. For a network defined by a graph G = (N, E), where N and E are number of nodes and edges respectively, the probability of degree distribution (P(k)) of the network is the ratio of the number of nodes having degree to the network size;

4 $${\boldsymbol{P}}({\boldsymbol{k}})=\frac{{{\boldsymbol{n}}}_{{\boldsymbol{k}}}}{{\boldsymbol{N}}}$$

Where, n_k_ is the number of nodes having degree k and N is the total number of nodes in the network. P(k) indicates the importance of hubs or modules in the network. It obeys power law P(k) ~k^−γ^ in scale-free and hierarchical networks depending on the value of γ which specifies the importance of hubs or modules in the network^20^.

#### Neighborhood connectivity

The number of neighbors of a node is considered as its connectivity. The neighborhood connectivity of a node n is defined as the average connectivity of all neighbors of n^21^. In the network (C_N_(k)) Neighborhood connectivity is given by,

5 $${{\boldsymbol{C}}}_{{\boldsymbol{N}}}({\boldsymbol{k}})={\sum }_{{\boldsymbol{q}}}{\boldsymbol{qP}}(\frac{{\boldsymbol{q}}}{{\boldsymbol{k}}})$$

where, ${\rm{P}}(\frac{q}{k})$ is the conditional probability that a link belonging to a node with connectivity k points to a node with connectivity q. The positive power dependence of C_N_(k) could be an indicator of assortivity in the network topology.

#### Clustering co-efficient

This topological parameter of a network represents the measure of the interconnection of a node with its neighborhood node and strength of its interconnection. It is the ratio of the number of its nearest neighborhood edges e_i_ to the total possible number of edges of degree k_i_. For an undirected network, clustering co-efficient (C(k_i_)) of ith node can be calculated by,

6 $${\boldsymbol{C}}({{\boldsymbol{k}}}_{{\boldsymbol{i}}})=\frac{2{{\boldsymbol{e}}}_{{\boldsymbol{i}}}}{{{\boldsymbol{k}}}_{{\boldsymbol{i}}}({{\boldsymbol{k}}}_{{\boldsymbol{i}}}-1)}$$

#### Betweenness centrality

Betweenness centrality C_B_ of a node represents the prominence of information flow in the network, and the extent to which the node has control over the other nodes in the network through communication^22,23^. If d_ij_ (v) indicates the number of geodesic paths from node i to node j passing through node v, and dij indicates number of geodesic paths from node i to j, then betweenness centrality (C_B_(v)) of a node v can be calculated by,

7 $${{\boldsymbol{C}}}_{{\boldsymbol{B}}}({\boldsymbol{v}})={\sum }_{{\boldsymbol{i}},{\boldsymbol{j}},{\boldsymbol{i}}\ne {\boldsymbol{j}}\ne {\boldsymbol{k}}}\frac{{{\boldsymbol{d}}}_{{\boldsymbol{ij}}}({\boldsymbol{v}})}{{{\boldsymbol{d}}}_{{\boldsymbol{ij}}}}$$

#### Closeness centrality

Closeness centrality (C_C_) measures how fast information is spread from a node to other nodes accessible from it in the network^24^. C_C_ of a node i is the reciprocal of the mean geodesic distance between the node and all other nodes connected to it in the network, and is given by,

8 $${{\boldsymbol{C}}}_{{\boldsymbol{C}}}({\boldsymbol{i}})=\frac{{\boldsymbol{n}}}{{\sum }_{{\boldsymbol{j}}}{{\boldsymbol{d}}}_{{\boldsymbol{ij}}}}$$

where d_ij_ represents the geodesic path length from nodes i to j, and n is the total number of vertices in the graph reachable from node i.

#### Eigenvector centrality

Eigenvector centrality of a node i (C_E_(i)) in a network is proportional to the sum of i’s neighbor centralities^25^, and it is given by,

9 $${{\boldsymbol{C}}}_{{\boldsymbol{E}}}({\boldsymbol{i}})=\frac{1}{{\boldsymbol{\lambda }}}\sum _{{\boldsymbol{j}}={\boldsymbol{nn}}({\boldsymbol{i}})}{{\boldsymbol{v}}}_{{\boldsymbol{j}}}$$

where, nn(i) indicates nearest neighbors of nodes i in the network. λ is eigenvalue of the eigenvector v_i_ is given by, Av_i_ = λv_i_ where, A is the adjacency matrix of the network (graph). The principal eigenvector of A, which corresponds to maximum eigenvalue λ_max_, is taken to have positive eigenvector centrality score. Eigenvector centrality can be used as an indicator of node’s spreading power in the network.

### Community detection/finding: Leading Eigen-vector method

The activities of the constructed network were defined at various levels of hierarchy to describe the modular nature, properties and the organizing principle of the hierarchical network. To detect the communities, the Leading Eigen Vector method (LEV)^26,27^ was used in R from package ‘igraph’^28^ in this study. The LEV method is the most promising one for community detection as it calculates the Eigen value for each link, exemplifying the significance of each link, not nodes. To obtain only motif, we detected modules from complete network and then sub-modules from the modules at each level of organization.

#### Modularity

Modularity is the measure of how fine a network is divided in communities^9^. Modularity (Q) is expressed as follows,

10 $${\boldsymbol{Q}}=\frac{1}{2{\boldsymbol{m}}}{\sum }_{{\boldsymbol{ij}}}({{\boldsymbol{A}}}_{{\boldsymbol{ij}}}-\frac{{{\boldsymbol{k}}}_{{\boldsymbol{i}}}{{\boldsymbol{k}}}_{{\boldsymbol{j}}}}{2{\boldsymbol{m}}}){\boldsymbol{\delta }}({{\boldsymbol{C}}}_{{\boldsymbol{i}}},{{\boldsymbol{C}}}_{{\boldsymbol{j}}})$$

where m is the total number of edges in the community, A_ij_ is the adjacency matrix of size i × j, k represents degrees, and the δ function yields 1 if nodes i and j are in the same community.

### Tracing of the Genes and Knock out Experiment

One particular challenge is to identify the main drivers that control the regulation of TS network. This was done through tracing of genes. This tracing of genes was performed up to motif level in various modules/sub-modules obtained from clustering. Through tracing the most significant and influential nodes within the network constructed was identified that regulates the network.

Further, the change in the organization of the network in the absence of these significant nodes was observed through the knockout experiment. The identified key regulators were successively removed from the constructed complete network, and the topological properties of the modified network were calculated again to describe the perturbations caused within the network due to the absence of these key regulators. The knockout experiment was repeated at different level of network organization to comprehend the role of these key genes in the network. The topological properties of the network were calculated using Network Analyzer, a plug-in in Cytoscape version 3.4, whereas for eigen value calculation, we used CytoNCA^29^, another plug-in in Cytoscape for topological properties.

### Local-community-paradigm (LCP) approach: Compactness of the network

LCP-Decomposition Plot (LCP-DP) is an approach to represent topological self-organisation as a local-community-paradigm (LCP), and consequently is used to visualise and examine the effect of LCP on network topology. It is a function of common neighbors (CN) index of interacting nodes and local community links (LCL) of each pair of interacting nodes in the network. It provides information on number, size and compactness of the communities in a given network^30^. The CN index between two nodes x and y can be calculated from the measure of overlapping between their sets of first-node-neighbors S(x) and S(y) given by, $CN=S(x)\cap S(y)$. If there is significant amount of overlapping between the sets S(x) and S(y) (large value of CN), the possible likelihood of interaction of these two nodes could happen and so an increase in CN represents the rise in compactness of the network, showing its faster information processing abilities. Further, the LCLs between the two nodes x and y, whose upper bound is defined by, $\max (LCL)=\frac{1}{2}CN(CN-1)$, is the number of internal links in local-community (LC), which is strongly inter-linked group of nodes. Most probably these two nodes link together if CN of these two nodes are members of LC^30^. LCP-DP has been found to have a linear dependence between CN and $\sqrt{LCL}$.

The LCP correlation (LCP-corr) is the Pearson correlation co-efficient between the variables CN and LCL defined by $LCP-corr=\frac{cov(CN,LCL)}{{{\rm{\sigma }}}_{CN}{{\rm{\sigma }}}_{LCL}}$ with CN > 1, where cov(CN, LCL) is the covariance between CN and LCL, σ_CN_ and σ_LCL_ are standard deviations of CN and LCL, respectively.

### Distribution of energy in the network: Hamiltonian energy calculation

At each level of the network, certain level of energy is maintained that helps organise the network at that level. This is measured by using Hamiltonian Energy (HE) of the network at that level/state within the formalism of Constant Potts Model^31,32^. HE gives the energy distribution not only at the global level of a network, but also at modular level, which is in the self-organization of the system. HE of a network or module or sub-module can be calculated by,

11 $${{\boldsymbol{H}}}^{[{\boldsymbol{c}}]}=-\,{\sum }_{{\boldsymbol{c}}}[{{\boldsymbol{e}}}_{{\boldsymbol{c}}}-{\boldsymbol{\gamma }}{{\boldsymbol{n}}}_{{\boldsymbol{c}}\,}^{2}]$$

Where e_c_ and n_c_ are the number of edges and nodes in a community ‘c’ and γ is the resolution parameter acting as an edge density threshold which is set to be 0.5.

### Predicting essential interactions by integrating the Orthology with PPI

Study has shown that there is a positive correlation between essentialities (essential proteins) and topological properties (centralities) of the proteins in PPI networks. As a consequence, a series of centrality measures based on network topological features have been used for identifying essential proteins, such as Degree Centrality, Betweenness Centrality, Closeness Centrality and Eigenvector Centrality. The proteins in the TS network were ranked in terms of their centrality (top 30 in each centrality). Then the ranking scores of these proteins were used to judge whether a protein is essential. Further the interacting partners of the hub proteins that fall into this category were identified.

In view of the facts that essential proteins are more evolutionarily conserved than non-essential proteins and they frequently bind each other, the prediction of essential proteins was done by integrating the orthology with PPI networks. To measure the conservation of the selected interacting proteins, their orthologous proteins in four different species namely, *Caenorhabditis elegans*, *Felis catus* (domestic cat), *Macaca mulatta* (macaque monkey), and *Homo sapien* were investigated (lower to higher level organisms). Information on orthologous proteins is taken from Version 8 of the InParanoid database^33^ (an ortholog database) which contains a collection of pairwise comparisons between 100 whole genomes (99 eukaryotes and 1 prokaryote) constructed by the INPARANOID program. Further the network of C. *elegans*, cat, macaque monkey, and human were constructed considering these 8 essential interacting proteins as hub genes for the analysis of the conserved interactions from STRING Protein Database^34^. STRING quantitatively integrates interaction data for a large number of organisms, and transfers information between these organisms where applicable.

## Electronic supplementary material

Supplementary Table S1 and S2
