# Supplementary material for: Assessment of the key regulatory genes and their Interologs for Turner Syndrome employing network approach
Source: Sci Rep. 2018 Jul 4;8:10091. doi: 10.1038/s41598-018-28375-0 (PMC6031616; doi:10.1038/s41598-018-28375-0)
Supplement: Supplementary file 1 — Supplementary Table S1 and S2 [file 41598_2018_28375_MOESM1_ESM.pdf]

Assessment of the key regulatory genes and their Interologs for Turner Syndrome employing network approach

Anam Farooqui[1], Safia Tazyeen[1], Mohd. Murshad Ahmed[1], Aftab Alam[1], Shahnawaz Ali[1], Md. Zubbair Malik[1], Sher Ali[1] and Romana Ishrat[1] *

[1]Centre for Interdisciplinary Research in Basic Sciences, Jamia Millia Islamia, New Delhi-110025, India

*Corresponding Author:

Dr Romana Ishrat (Assistant Professor)

Centre for Interdisciplinary Research in Basic Sciences,

Jamia Millia Islamia, New Delhi-110025, India.

Email address – romana05@gmail.com

Table S1. List of top 120 Proteins identified by centrality-based method in TS network

S.N.	Protein Name (Degree Distribution)	Protein Name (Betweenness Centrality)	Protein Name (Closeness Centrality)	Protein Name (Eigen Value)
1	HSP7C	EP300	Polyubiquitin-C	EF1A1
2	EF1A1	CALM	EP300	YBOX1
3	CALM	ANDR	ANDR	GEMI4
4	YBOX1	SMAD2	SMAD2	MPCP
5	Polyubiquitin-C	JAK2	CALM	FILA2
6	MPCP	SMAD4	SMAD4	GEMI2
7	GEMI4	HSP7C	ESR1	CALM
8	EP300	HDAC3	HSP7C	FLNA
9	TBA4A	CTNB1	HDAC3	TBA4A
10	ALBU	Polyubiquitin-C	HS90A	HSPB1
11	DCD	PTN1	YBOX1	RO52
12	IGHG1	INSR	EF1A1	SERPH
13	FLNA	TGFR2	MYC	ANXA2
14	GEMI2	XIAP	CTNB1	HSP7C
15	FILA2	TGFR1	NCOR1	IGHG1
16	ACTB	HS90A	P53	DCD
17	SMAD4	VDR	MAX	ALBU
18	HSPB1	NCOA3	RUNX1	MYH9
19	G3P	NHRF2	PRKDC	ACTB
20	MYH9	EF1A1	IF2B3	G3P
21	RS3	IGF1R	INSR	ATPA
22	ANXA2	Mitogen-activated protein kinase 11	FLNA	HS90A
23	SERPH	SRC	TGFR1	Polyubiquitin-C
24	RO52	ESR1	Nucleophosmin	RL13
25	SMAD2	PTN11	JAK2	IF2B3
26	ATPA	T-DHT/AR	PTN11	40S ribosomal protein S4
27	IF2B3	Adapter protein GRB2	PELP1	RS3
28	RL13	ZFYV9	Histone H4	EP300
29	40S ribosomal protein S4	CTND1	NCOA3	SMAD2
30	HS90A	NCOR1	MBB1A	SMAD4

Table S2. Orthologous proteins of *C. elegans, F. Catus and M. mulatta*

**Protein Name**	**Orthologous Protein/Gene**		
H. sapiens	***C. elegans***	***F. catus***	***M. mulatta***
SRY	SOX-3	SRY	SRY
HDAC3	HDA-3	HDAC3	HDAC3
RPS4Y1	-	-	RPS4Y1
RPS3	RPS4	RPS3	RPS3
KDM6A	UTX-1	KDM6A	KDM6A
WDR5	WDR-5.1	WDR5	WDR5
ASH2L	ASH-2	ASH2L	ASH2L
BDNF	-	BDNF	BDNF
MBPT1	-	MBTPS1	MBTPS1
CAPS2	UNC-31	CADPS2	CADPS2
CBPE	EGL-21	CPE	-
NOS3	R05G6.4	NOS3	NOS3
